# Neurosteroid [3α,5α]-3-hydroxy-pregnan-20-one enhances IL-10 production via endosomal TRIF-dependent TLR4 signaling pathway

**DOI:** 10.3389/fendo.2023.1299420

**Published:** 2023-12-21

**Authors:** Irina Balan, Adelina Grusca, Todd K. O’Buckley, A. Leslie Morrow

**Affiliations:** ^1^ Bowles Center for Alcohol Studies, University of North Carolina at Chapel Hill, Chapel Hill, NC, United States; ^2^ Department of Psychiatry, University of North Carolina at Chapel Hill, Chapel Hill, NC, United States; ^3^ Department of Pharmacology, University of North Carolina at Chapel Hill, Chapel Hill, NC, United States

**Keywords:** [3α,5α]-3-hydroxy-pregnan-20-one (3α,5α-THP), [3α,5α]-3,21-dihydroxypregnan-20-one (3α,5α-THDOC), toll-like receptor 4, toll/interleukin-1 receptor domain-containing adapter-inducing interferon-β (TRIF), neuroinflammation

## Abstract

**Background:**

Previous studies demonstrated the inhibitory effect of allopregnanolone (3α,5α-THP) on the activation of inflammatory toll-like receptor 4 (TLR4) signals in RAW264.7 macrophages and the brains of selectively bred alcohol-preferring (P) rats. In the current study, we investigated the impact of 3α,5α-THP on the levels of IL-10 and activation of the TRIF-dependent endosomal TLR4 pathway.

**Methods:**

The amygdala and nucleus accumbens (NAc) of P rats, which exhibit innately activated TLR4 pathways as well as RAW264.7 cells, were used. Enzyme-linked immunosorbent assays (ELISA) and immunoblotting assays were used to ascertain the effects of 3α,5α-THP on the TRIF-dependent endosomal TLR4 pathway and endosomes were isolated to examine translocation of TLR4 and TRIF. Additionally, we investigated the effects of 3α,5α-THP and 3α,5α-THDOC (0.1, 0.3, and 1.0 µM) on the levels of IL-10 in RAW264.7 macrophages. Finally, we examined whether inhibiting TRIF (using TRIF siRNA) in RAW264.7 cells altered the levels of IL-10.

**Results:**

3α,5α-THP administration facilitated activation of the endosomal TRIF-dependent TLR4 pathway in males, but not female P rats. 3α,5α-THP increased IL-10 levels (+13.2 ± 6.5%) and BDNF levels (+21.1 ± 11.5%) in the male amygdala. These effects were associated with increases in pTRAM (+86.4 ± 28.4%), SP1 (+122.2 ± 74.9%), and PI(3)K-p110δ (+61.6 ± 21.6%), and a reduction of TIRAP (−13.7 ± 6.0%), indicating the activation of the endosomal TRIF-dependent TLR4 signaling pathway. Comparable effects were observed in NAc of these animals. Furthermore, 3α,5α-THP enhanced the accumulation of TLR4 (+43.9 ± 11.3%) and TRIF (+64.8 ± 32.8%) in endosomes, with no significant effect on TLR3 accumulation. Additionally, 3α,5α-THP facilitated the transition from early endosomes to late endosomes (increasing Rab7 levels: +35.8 ± 18.4%). In RAW264.7 cells, imiquimod (30 µg/mL) reduced IL-10 while 3α,5α-THP and 3α,5α-THDOC (0.1, 0.3, and 1.0 µM) restored IL-10 levels. To determine the role of the TRIF-dependent TLR4 signaling pathway in IL-10 production, the downregulation of TRIF (−62.9 ± 28.2%) in RAW264.7 cells led to a reduction in IL-10 levels (−42.3 ± 8.4%). TRIF (−62.9 ± 28.2%) in RAW264.7 cells led to a reduction in IL-10 levels (−42.3 ± 8.4%) and 3α,5α-THP (1.0 µM) no longer restored the reduced IL-10 levels.

**Conclusion:**

The results demonstrate 3α,5α-THP enhancement of the endosomal TLR4-TRIF anti-inflammatory signals and elevations of IL-10 in male P rat brain that were not detected in female P rat brain. These effects hold significant implications for controlling inflammatory responses in both the brain and peripheral immune cells.

## 1 Introduction

In recent years, there has been growing recognition of the critical role played by peripheral and brain inflammation in the development and progression of neurological and psychiatric disorders (1, 2). In this context, endogenous pregnane neurosteroids, including pregnenolone and allopregnanolone([3α,5α]3-hydroxypregnan-20-one; 3α,5α-THP), have emerged as promising regulators of inflammatory and neuroinflammatory processes (3–11).

Neuroactive steroids, synthesized in the brain and peripheral tissues from cholesterol, play diverse and vital roles in governing various physiological functions, including stress responses, mood and alcohol response, and immune regulation. Furthermore, some neuroactive steroids exhibit remarkable ability to modulate neurotransmitter systems in the brain, notably gamma-aminobutyric acid (GABA) and glutamate. Functioning as a positive allosteric modulator of GABA_A_ receptors, 3α,5α-THP and [3α,5α]-3,21-dihydroxypregnan-20-one (tetrahydrodeoxycorticosterone; 3α,5α-THDOC) enhance inhibitory neurotransmission and thereby counterbalance excitatory responses (12–14). The GABAergic properties of these neurosteroids contribute to anxiolysis, sedation, anti-depressant activity, anti-convulsant activity, and the augmentation of inhibitory circuits within the brain (14–16). However, it is important to note that anti-inflammatory effects of pregnenolone and 3α,5α-THP are found to be independent of its GABAergic mechanisms (6, 10).

In our previous studies, we demonstrated that 3α,5α-THP exerts inhibitory effects on inflammatory TLR4 signals and the production of inflammatory cytokines and chemokines by disrupting the binding of TLR4 with the myeloid differentiation factor 2 (MD2) or myeloid differentiation primary response 88 (MyD88) in both mouse and human macrophages, as well as in the brains of P rats (6, 7, 10). TLR4 activation triggers a cascade of protein–protein interactions that lead to the activation (phosphorylation) of transcription factors, subsequently translocating to the nucleus and facilitating the production of proinflammatory cytokines and chemokines, which are elevated in various disease states (17–23). TLR4 can be activated through two distinct signaling pathways. The MyD88-dependent pathway operates on the plasma membrane and involves the recruitment of the co-receptor MD2 and adaptor molecules MyD88 and toll/interleukin-1 receptor domain containing adaptor protein (TIRAP) (18, 23). On the other hand, the toll/interleukin-1 receptor domain-containing adapter-inducing interferon-β (TRIF)-dependent pathway occurs in endosomes and is initiated by adaptors TRIF and TLR4-specific TRIF-related adapter molecule (TRAM) (24–26). Apart from TLR4, TLR3 operates exclusively within endosomes and depends solely on the TRIF-dependent pathway (26). While 3α,5α-THP has been found to specifically inhibit the MyD88-dependent TLR4 pro-inflammatory signals, it does not affect TRIF-dependent pro-inflammatory signals (7).

However, it is crucial to emphasize that TLR4 activation is a two-sided phenomenon, capable of initiating not only inflammatory signals but also the anti-inflammatory mediator cytokine interleukin-10 (IL-10) and the neuroprotective modulator brain-derived neurotrophic factor (BDNF) (27, 28). Specifically, the transition of TLR4 from the plasma membrane to endosomes is associated with the activation of the p110δ isoform of phosphatidylinositol-3-OH kinase (PI(3)K), leading to the production of the IL-10. Simultaneously, this activation leads to the degradation of TIRAP, effectively reducing TIRAP-MyD88-mediated pro-inflammatory signaling (27, 29). In addition, TLR4 activation indirectly participates in the production of BDNF. Suppression of TLR4/nuclear factor kappa B inflammatory signaling leads to the activation of associated cyclic adenosine monophosphate response element-binding protein (CREB) signaling, which, in turn, enhances the expression of BDNF in brain tissues (28). Moreover, some studies have suggested that BDNF may modulate the production and release of IL-10 (30–32), highlighting a potential link between neurotrophic and anti-inflammatory signaling pathways. In the realm of psychiatric and neurodegenerative disorders, IL-10 and BDNF emerge as impactful anti-inflammatory agents with significant therapeutic implications. Their multifaceted roles extend beyond traditional neurobiology, paving the way for novel interventions and management strategies for a variety of inflammation-associated neurological conditions (32–35).

In this study, our objective was to explore the impact of 3α,5α-THP on the activation of the TRIF-dependent endosomal TLR4 signaling pathway and IL-10 and BDNF expression in both male and female amygdala and NAc. We used P rats for these studies as they represent a model of innate TLR activation where the effects on brain TLR pathways can be examined in the absence of peripheral immune activation (7, 36). Additionally, we investigated the effects of different concentrations of 3α,5α-THP and 3α,5α-THDOC on IL-10 levels in RAW264.7 macrophages and examined the role of the TRIF-dependent TLR4 pathway by downregulating TRIF in these cells.

## 2 Materials and methods

### 2.1 Animals

Male and female P rats (males: *N* = 60; females: *N* = 20) aged 3–4 months and weighing between 250 and 550 g, were procured from the Alcohol Research Center at Indiana University School of Medicine and bred in-house at the University of North Carolina (NC) School of Medicine. These rats were housed in pairs in Plexiglas cages containing corn cob bedding, maintained on a 12-h light–dark cycle (light onset at 0700 h), and given food and water *ad libitum*. Prior to experimentation, rats were acclimated to handling and saline injection for 1 week. The study focused on investigating the regulation of neuroimmune signaling by 3α,5α-THP in P rats. This was motivated by the observation that selective breeding for alcohol preference resulted in the innate activation of the pro-inflammatory TLR4 and TLR7 pathways in brain regions of P rats, including the amygdala and NAc (6, 7, 36). The amygdala and NAc are implicated in neuroimmune responses via TLR pathways in various neuropathological conditions, including alcohol use disorders (36–41). Furthermore, P rats exhibit other pathological behavioral traits, including impulsivity (42), anxiety-like behavior (43, 44), and stress reactivity (45). The experimental procedures adhered to NIH Guidelines and were approved by the Institutional Animal Care and Use Committee at the University of North Carolina, School of Medicine. To mitigate potential circadian variations in neurosteroid levels, all experiments were conducted in the morning (46, 47). For experimentation, P rats were randomly assigned to receive either 3α,5α-THP (10 mg/kg) (males: *N* = 20; females: *N* = 10) or vehicle (45% w/v 2-hydroxypropyl-β-cyclodextrin) (males: *N* = 20; females: *N* = 10) through intraperitoneal (IP) injection. In addition, male P rats received IP injections of either 3α,5α-THDOC (10 mg/kg) (*N* = 10) or a vehicle solution (45% w/v 2-hydroxypropyl-β-cyclodextrin) (*N* = 10). They were sacrificed after 60 min. Euthanasia was performed by decapitation, and the brains were collected and stored at −80°C. Coronal brain sections were initially obtained using a cast aluminum cutting block. Subsequently, the amygdala and NAc were meticulously separated from these slices, guided by well-established neuroanatomical landmarks, as previously described by Heffner et al. (1980) (48).

The selection of the time point and dosage was guided by prior research indicating that higher doses of 3α,5α-THP exhibit sedative effects, while demonstrating anticonvulsant and anxiolytic properties, without inducing hypnotic effects (49–51). Additionally, our earlier works have established the inhibitory impact of 3α,5α-THP on inflammatory TLR pathway activation in the P rat brain (6, 7).

### 2.2 Cell culture and reagents

Mouse macrophage/monocyte (RAW264.7) cells were procured from the American Type Culture Collection (Manassas, VA). They were cultured in Dulbecco’s modified Eagle’s medium (DMEM) from Gibco (Gaithersburg, MD), supplemented with 10% fetal bovine serum (FBS) obtained from Gemini (West Sacramento, CA) and 1% penicillin/streptomycin 100× from Gibco. The cells were maintained at 37°C in a 5% CO_2_ humidified atmosphere.

A day before the experiment, RAW264.7 cells were seeded in T-25 tissue culture flasks, with a density of 0.7×10^6^ cells per flask. On the following day, the cells were exposed to vehicle or imiquimod (30 μg/mL) obtained from InvivoGen (Cat. #tlrl-imqs, San Diego, CA) in DMEM (without FBS and antibiotics) for 4 h. Subsequently, the imiquimod solution was substituted with 3α,5α-THP (at concentrations of 0.1, 0.3, and 1.0 μM), 3α,5α-THDOC (at concentrations of 0.1, 0.3, 1.0 μM), or a control solution containing 0.05% dimethyl sulfoxide (DMSO) in DMEM (without FBS and antibiotics) for 20 h. The stock solutions of the neurosteroids (2 mM) were prepared using DMSO, leading to a DMSO concentration of 0.05% within the 1.0 μM neurosteroid solution.

### 2.3 siRNA transfection

Silencer^®^ pre-designed mouse TRIF/TICAM-1 siRNA (60 pmol) (siRNA ID# 173696, P/N AM16704, Lot# ASO2L8L4, Ambion by Life Technologies, Carlsbad, CA) and Silencer^®^ Negative Control #4 siRNA (60 pmol) (Cat. # AM4641, Ambion by Life Technologies) were both diluted in 500 µL of Opti-MEM^®^ I Medium (without serum) within the wells of a six-well tissue culture plate and gently mixed. Lipofectamine™ RNAiMAX (5 µL) (Cat. # 13778030, Thermo Fisher Scientific, Waltham, MA) was introduced into each well containing the diluted siRNA molecules. The contents were gently mixed and incubated for 15 min at room temperature (RT). RAW264.7 cells were diluted in DMEM medium (Gibco), supplemented with 10% FBS (Gemini) (without antibiotics). Subsequently, 2,495 µL of the diluted cells (0.2×10^6^ cells/well) were added to each well containing the siRNA–Lipofectamine™ RNAiMAX complexes. This action led to a final volume of 3,000 µL and a final siRNA concentration of 20 nM. The cells were then placed in a 37°C incubator (5% CO_2_) overnight. The following day, the medium was replaced with DMEM medium (Gibco), supplemented with 10% FBS (Gemini) (without antibiotics). The cells were incubated further for a total duration of up to 72 h. The cells were collected and subjected to immunoblotting to assess the levels of TRIF and IL-10. There was an additional experiment where after overnight incubation with siRNA complexes, the medium was replaced with either imiquimod (30 μg/mL) or a mock control solution in DMEM (without FBS and antibiotics) for 4 h. Subsequently, both the imiquimod and mock control solutions were substituted with 3α,5α-THP (1.0 μM) or a control solution containing 0.05% DMSO, and the experiment continued for a total duration of 72 h. The collected cells were then subjected to an enzyme-linked immunosorbent assay (ELISA) to measure the levels of IL-10.

### 2.4 Cell lysate preparation for immunoblotting and ELISA

For immunoblotting and ELISA of whole tissue lysates, the amygdala and NAc tissues were dissected and lysed using CelLytic MT (dialyzable mild detergent, bicine, and 150 mM NaCl) from Sigma Aldrich (St. Louis, MO, USA, Cat. # C3228), along with protease and phosphatase inhibitor cocktails (Sigma). RAW264.7 cells were lysed using radioimmunoprecipitation (RIPA) buffer from Sigma (Cat. # R0278), supplemented with protease and phosphatase inhibitor cocktails (Sigma). The lysates were sonicated twice for 30 s at 25% output power using a Sonicator ultrasonic processor from Misonix, Inc. (Farmingdale, NY), followed by centrifugation at 14,000 × g and 4°C for 30 min. Total protein levels were quantified using the bicinchoninic acid (BCA) assay from Thermo Fisher Scientific (Waltham, MA, USA, Cat.# 23228 and Cat.# 1859078).

### 2.5 Immunoblotting

The protein samples (35 μg/lane) were subjected to denaturation at 95°C for 5 min using lithium dodecyl sulfate (LDS) sample buffer (Cat.# NP0007, Thermo Fisher Scientific) along with sample reducing agent (Cat.# NP0009, Thermo Fisher Scientific). The proteins were separated through electrophoresis on NuPAGE™ 10% Bis-Tris Midi Protein Gel (Cat.# WG1202 and WG1203, Thermo Fisher Scientific), with an initial voltage of 125 V for 10 min followed by 165 V for the remaining duration. Electrophoretically separated samples were translocated onto a polyvinylidene difluoride membrane (PVDF; Cat.# 1620177, Bio-Rad). The membranes underwent a blocking step of 2 h at RT utilizing either a 5% solution of blotting-grade blocker (Cat.# #1706404, Bio-Rad) or 5% bovine serum albumin (BSA) (in the case of phosphorylated primary antibodies). Following the blocking step, the membranes underwent an incubation period overnight (at 4°C) with primary antibodies, followed by a 1h exposure to horseradish peroxidase-conjugated secondary antibodies at RT. Both primary and secondary antibodies were appropriately diluted in either a 5% blotting-grade blocker buffer or 5% BSA (for phosphorylated primary antibodies). Following the antibody incubation steps, the membranes underwent triple washing in Tris-buffered saline supplemented with 0.05% Tween-20 (TNT), each wash lasting 10 min. Immunoreactive bands were visualized using the PlusECL kit reagents (Cat.# NEL105001EA, Perkin Elmer, Waltham, MA), and subsequent chemiluminescent signal detection was performed using the ImageQuant LAS4000 system (GE Healthcare, Amersham, UK). Membrane images were analyzed using ImageQuant TL version 8.1.0.0 software. Normalization of each densitometric measurement occurred through division by the corresponding β-actin densitometric measurement. The obtained values are presented as percentages in relation to the average value of the vehicle control, along with the corresponding standard error of the mean (SEM).

### 2.6 ELISA

ELISAs were performed on protein extracts using ELISA kits (Raybiotech, Norcross, GA) designed for IL-10 (Cat. # ELR-IL10-1), following the manufacturer’s guidelines. The outcomes are presented in picograms per milligram of total protein (pg/mg).

### 2.7 Antibodies

Antibodies were procured commercially and utilized according to the manufacturer’s instructions following validation. Details of primary antibodies, host species, clonality, and dilutions are provided in **Table S1**. Horseradish peroxidase-labeled secondary antibodies were anti-rabbit (Cat. # 7074, Cell Signaling Technology) and anti-mouse (Cat# 7076, Cell Signaling Technology).

### 2.8 Endosome isolation

Endosomes were isolated from the amygdala of male P rats that received IP injections of either 3α,5α-THP (10 mg/kg; 60 min) or vehicle (45% w/v 2-hydroxypropyl-β-cyclodextrin) (*N* = 10/group). The isolation procedure followed the manufacturer’s instructions utilizing the Minute™ Endosome Isolation and Cell Fractionation Kit (Cat. # ED-028, Invent Biotechnologies, Plymouth, MN). In summary, the frozen brain tissue (~50–80 mg) was placed within filter cartridges and ground using 300 µL of buffer A (containing protease and phosphatase inhibitors) for 1 min on ice. Subsequently, 200 µL of buffer A containing protease and phosphatase inhibitors was added to the tissue homogenate, which was then incubated for 5 min on ice. The homogenate underwent two centrifugation steps at 16,000 × *g* for 30 s each. The resulting pellet was resuspended and subjected to centrifugation at 700 × *g* for 2.5 min, with this pellet containing intact nuclei and some unruptured cells. The collected supernatant was centrifuged at 4°C for 60 min at 16,000 × *g*. In this phase, the pellet primarily comprised larger organelles and plasma membranes. The supernatant obtained from the previous centrifugation was combined with buffer B (buffer B to supernatant ratio: 1:2), and the mixture was then incubated at 4°C overnight. Subsequently, the mixture was centrifuged at 10,000 × *g* for 30 min at 4°C. The resulting supernatant contained the cytosolic fraction. The pellet containing isolated endosomes was resuspended in MinuteTM Denaturing Protein Solubilization Reagent (Cat # WA-009), along with protease and phosphatase inhibitors. To ascertain the total protein levels, the BCA assay was employed (Cat.# 23228, 1859078, Thermo Fisher Scientific). The protein samples (50 μg/lane) were denatured by heating at 95°C for 5 min using LDS sample buffer (Cat.# NP0007, Thermo Fisher Scientific) supplemented with a sample reducing agent (Cat.# NP0009, Thermo Fisher Scientific). After denaturation, the proteins underwent separation through electrophoresis by employing a NuPAGE™ 10% Bis-Tris Midi Protein Gel (Thermo Fisher Scientific). Subsequently, immunoblotting was conducted according to the previously described procedure. The outcomes were normalized by the total protein levels. The total protein levels were visualized by utilizing the No-StainTM Protein Labeling Reagent (Cat.# A44449, Thermo Fisher Scientific) following the manufacturer’s guidelines. The results are expressed as % ± SEM of the vehicle controls. We confirmed the enrichment of endosomes in the endosomal fraction through immunoblotting using the endosomal marker early endosomal antigen 1 (EEA1). Negative controls included the nucleus, plasma membrane, and cytosolic fractions. Significantly, only the endosomal fractions showed detectable EEA1-positive immunoblotting bands (data not shown).

### 2.9 Statistics

Studies were conducted independently in male and female amygdala and NAc. We conducted comparisons within each P rat experimental group to assess the differences (%) in the levels of proteins of interest between neurosteroid treatment and vehicle control treatment. Each experimental group consisted of two datasets (vehicle vs. neurosteroid), allowing us to apply either a parametric *t*-test or a nonparametric Mann–Whitney *U* test, depending on whether the data followed a normal (Gaussian) distribution, as determined by the Shapiro–Wilk normality test. For normally distributed data, we utilized an unpaired *t*-test. We examined the resulting *t*-value, degrees of freedom (df), and significance level (*p*-value). In cases where the data did not pass the normality test, we employed the Mann–Whitney *U* test. This involved analyzing the resulting *U*-value, sample size (*n*), and *p*-value. To assess the influence of different concentrations of 3α,5α-THP and 3α,5α-THDOC on IL-10 expression in imiquimod-activated RAW264.7 cells, a one-way ANOVA followed by Fisher’s test was employed. This allowed for the comparison of IL-10 differences (%) between the imiquimod-activated cells and the non-activated (control) cells, as well as between the imiquimod-activated cells and both the imiquimod-activated and neurosteroid-treated cells. The ANOVA results, including *F*-values (with degrees of freedom DFn, DFd) and corresponding *p*-values, were examined. Additionally, *p*-values and *n*-values for the Fisher’s test were reported. Statistical analysis was carried out using GraphPad Prism 9.4.1 software. A significance level of *p* < 0.05 was adopted to determine statistical significance.

## 3 Results

### 3.1 3α,5α-THP upregulates IL-10 and BDNF levels in male but not female amygdala and NAc of P rats

**Figure 1:**
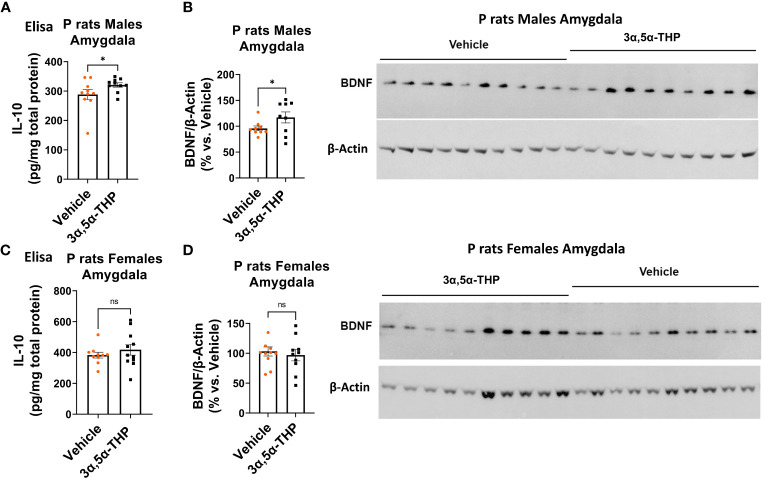
3α,5α-THP elevates IL-10 and BDNF levels in the amygdala of male P rats. 3α,5α-THP (10 mg/kg, IP) or a vehicle control was administered to male and female P rats, and the levels of IL-10 and BDNF were determined within the amygdala. **(A)** In male P rats, administration of 3α,5α-THP led to a significant increase in IL-10 levels in the amygdala (+13.2 ± 6.5%; *t*-test: *t* = 1.77, df = 18, *n* = 10, *p* = 0.047). **(B)** Similarly, a significant elevation in BDNF levels was observed in the amygdala of male P rats following 3α,5α-THP treatment (+21.1 ± 11.5%; *t*-test: *t* = 1.84, df = 18, *n* = 10, *p* = 0.04). **(C)** In contrast, female P rats did not exhibit a significant change in IL-10 expression in the amygdala following 3α,5α-THP treatment (Mann–Whitney test: *U* = 50.5, *n* = 10, *p* = 0.39). **(D)** Additionally, there were no significant alterations in the levels of BDNF in the amygdala of female P rats after 3α,5α-THP administration (*t*-test: *t* = 0.52, df = 18, *n* = 10, *p* = 0.61). *p < 0.05.

**Figure 2:**
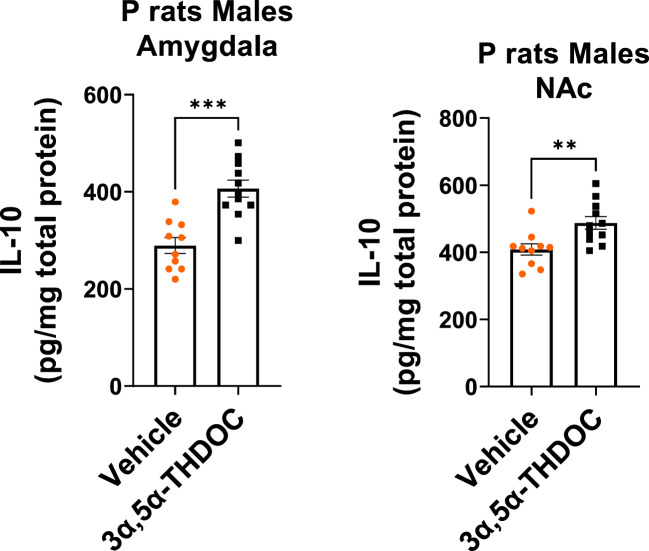
3α,5α-THDOC elevates IL-10 levels in the amygdala and NAc of male P rats. 3α,5α-THDOC (10 mg/kg, IP) or a vehicle control was administered to male P rats, and the levels of IL-10 were determined within the amygdala and NAc. The administration of 3α,5α-THDOC led to an increase in IL-10 levels in the amygdala (+40.4 ± 8.3%; *t*-test: *t* = 4.85, df = 18, *n* = 10, *p* = 0.0001) and NAc (+19.3 ± 6.3%; *t*-test: *t* = 3.09, df = 18, *n* = 10, *p* = 0.006). **p < 0.01, ***p < 0.001.

Since TLR4 is involved in both the generation of inflammatory mediators (17, 18, 21, 52) and the upregulation of anti-inflammatory mediators and trophic factors (27–29), we further investigated the impact of 3α,5α-THP (10 mg/kg, IP) on the levels of IL-10 and BDNF in the amygdala and NAc of male and female P rats. We also examined the effects of 3α,5α-THDOC on the levels of IL-10 in the male amygdala and NAc. In males, the administration of 3α,5α-THP led to a statistically significant increase in IL-10 levels in the amygdala (+13.2 ± 6.5%; *t*-test *p* = 0.047) (**Figure 1A**) and NAc (+12.8 ± 5.9%; *t*-test *p* = 0.04) (**Figure S1A**). Similarly, following 3α,5α-THP treatment, there were statistically significant elevations in BDNF levels in both the amygdala (+21.1 ± 11.5%; *t*-test *p* = 0.04) (**Figure 1B**) and the NAc (+34.2 ± 11.2%; Mann–Whitney test *p* = 0.01) (**Figure S1B**). In contrast, 3α,5α-THP treatment of females did not exert a significant effect on IL-10 expression in either the amygdala (**Figure 1C**) or the NAc (**Figure S1C**). Additionally, there were no changes observed in the levels of BDNF in females, either in the amygdala (**Figure 1D**) or in the NAc (**Figure S1D**) following 3α,5α-THP treatment. In males, the administration of 3α,5α-THDOC led to an increase in IL-10 levels in the amygdala (+40.4 ± 8.3%; *t*-test *p* = 0.0001) and NAc (+19.3 ± 6.3%; *t*-test *p* = 0.006) (**Figure 2**).

These findings highlight the sex-specific effects of 3α,5α-THP on IL-10 and BDNF expression in the P rat brain, suggesting that its impact on IL-10 and BDNF may be influenced by sex-dependent mechanisms.

### 3.2 Sex-specific effects of 3α,5α-THP on TRAM-dependent TLR4/TRIF signaling in the P rat brain: activation in males, inhibition in females

Existing data suggest that TLR4/TRIF signaling may exert anti-inflammatory and neuroprotective effects, leading to the upregulation of anti-inflammatory type I interferon-associated genes, as well as IL-10 and BDNF (27–29, 53). Because our findings demonstrated an increase in IL-10 and BDNF levels exclusively in male P rat brains, we investigated potential sex-specific responses of 3α,5α-THP on TLR4/TRIF signaling activation in the P rat brain. To investigate potential sex differences in TLR4/TRIF signaling, we measured the levels of phosphorylated (p) or activated TRAM, which serves as a specific marker for TLR4/TRIF pathway activation (25).

**Figure 3:**
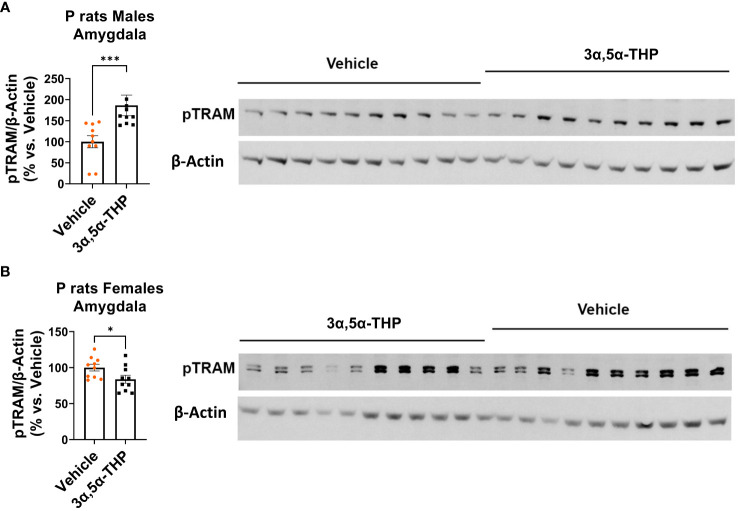
Sex-specific effects of 3α,5α-THP on TRAM-dependent TLR4-TRIF signaling in the amygdala of P rats: activation in males, inhibition in females. After 3α,5α-THP (10 mg/kg, IP) or a vehicle administration to male and female P rats, we analyzed the levels of pTRAM within the amygdala. **(A)** In males, 3α,5α-THP administration led to a significant increase in pTRAM levels (+86.4 ± 28.4%; Mann–Whitney test: *U* = 8, *n* = 10, *p* = 0.0007), whereas **(B)** females exhibited inhibited pTRAM levels (−16.2 ± 7.2%; *t*-test: *t* = 2.24, df = 18, *n* = 10, *p* = 0.04). *p < 0.05; ***p < 0.001.

We found that in males, the administration of 3α,5α-THP led to a significant increase in pTRAM levels in the amygdala (+86.4 ± 28.4%; Mann–Whitney *p* = 0.0007) (**Figure 3A**) and the NAc (+22.9 ± 7.5%; *t*-test *p* = 0.007) (**Figure S2A**). By contrast, in females, pTRAM levels were reduced in both the amygdala (−16.2 ± 7.2%; *t*-test *p* = 0.04) (**Figure 3B**) and the NAc (−12.1 ± 6.3%; *t*-test *p =* 0.03) (**Figure S2B**). Additionally, in females, TRIF levels were inhibited in the amygdala (−14.0 ± 6.9%, *t*-test *p* = 0.049) and the NAc (−17.0 ± 6.2%, *t*-test *p* = 0.04) (**Table S2**). However, in males, TRIF levels were not changed in both the amygdala and NAc (**Table 1**). The data demonstrate that 3α,5α-THP promotes activation of the TRAM-dependent TLR4/TRIF pathway in the P rat brain of males, but inhibits this pathway in female P rat brain.

**Table 1:** Evaluated signaling pathway members that demonstrated no response to 3α,5α-THP or they were inhibited by 3α,5α-THP in the whole tissue lysates of the amygdala and nucleus accumbens (NAc) of male P rats.

Pathway members	3α,5α-THP vs. Vehicle *t*-test (*t*, df, *p*)/Mann–Whitney test: *U*, *p*, *n*	
	Amygdala	NAc
TLR4	No difference *t* = 1.55, df = 18, *p* = 0.07	No difference *t* = 1.84, df = 18, *p* = 0.08
TRIF	No difference *t* = 1.68, df = 18, *p* = 0.11	No difference *t* = 0.98, df = 18, *p* = 0.34
c-Maf	No difference *t* = 0.65, df = 18, *p* = 0.52	No difference *t* = 0.42, df = 18, *p* = 0.68
pAkt	Inhibition: −34.2 ± 26.4% *U* = 26, *p* = 0.04*, *n* = 10	Inhibition: −20.7 ± 3.5% *U* = 22, *p* = 0.02*, *n* = 10
HSP70	No difference *U* = 35.5, *p* = 0.29, *n* = 10	Inhibition: −12.4 ± 5.1% *t* = 2.26, df = 18, *p* = 0.04*
pCREB	No difference *t* = 1.01, df = 18, *p* = 0.16	Inhibition: -39.0 ± 2.4% *U* = 11.5, *p* = 0.002**, *n* = 10

*p < 0.05, **p < 0.01.

### 3.3 Upregulation of SP1 and p110δ-PI(3)K as well as TIRAP reduction were observed in both male and female P rat brains

Next, we evaluated the levels of the transcription factor SP1, which is known to play a role in IL-10 production (54). Additionally, we investigated the activation of p110δ-PI(3)K, a component associated with anti-inflammatory TLR4/TRIF signaling that facilitates the degradation of TIRAP, an essential adaptor involved in inflammatory TLR4/MyD88 signaling (27, 29).

**Figure 4:**
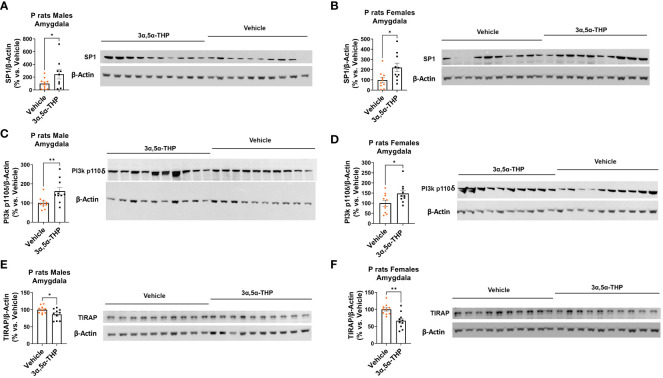
3α,5α-THP upregulates SP1 and p110δ-PI(3)K levels and reduces TIRAP levels in the amygdala of male and female P rats. 3α,5α-THP (10 mg/kg, IP) or a vehicle control was administered to male and female P rats to assess its impact on SP1, p110δ-PI(3)K, and TIRAP levels within the amygdala. 3α,5α-THP elevates SP1 levels in the amygdala of both **(A)** male (+122.2 ± 74.9%; *t*-test: *t* = 1.94, df = 18, *n* = 10, *p* = 0.03) and **(B)** female (+122.3 ± 49.4%; *t*-test: *t* = 2.48, df = 18, *n* = 10, *p* = 0.01) P rats. 3α,5α-THP upregulates p110δ-PI(3)K levels in the amygdala of both **(C)** male (+61.6 ± 21.6%; Mann–Whitney test: *U* = 16, *n* = 10, *p* = 0.009) and **(D)** female (+47.8 ± 21.1%; *t*-test: *t* = 2.66, df = 18, *n* = 10, *p* = 0.02) P rats. 3α,5α-THP reduces TIRAP levels in the amygdala of both **(E)** male (−13.7 ± 6.0%; *t*-test: *t* = 2.26, df = 18, *n* = 10, *p* = 0.02) and **(F)** female (−33.2 ± 8.9%; *t*-test: *t* = 3.71, df = 18, *n* = 10, *p* = 0.002) P rats. *p < 0.05; **p < 0.01.

We observed increased levels of SP1 in both male [amygdala: +122.2 ± 74.9%; *t*-test *p* = 0.03 (**Figure 4A**); NAc: +47.6 ± 27.5%; *t*-test *p* = 0.049 (**Figure S3A**)] and female [amygdala: +122.3 ± 49.4%; *t*-test *p* = 0.01 (**Figure 4B**); NAc: +254.3 ± 134.9%; Mann–Whitney test *p* = 0.02 (**Figure S3B**)] P rat brains following 3α,5α-THP treatment. The observation that increases in SP1 were found in both male and female P rat brain suggests that the transcription factor SP1 may play a broader role beyond regulating IL-10 expression.

3α,5α-THP significantly upregulated p110δ-PI(3)K levels in both male [amygdala: +61.6 ± 21.6%; Mann–Whitney test *p* = 0.009 (**Figure 4C**); NAc: +47.3 ± 20.6%; Mann–Whitney test *p* = 0.03 (**Figure S3C**)] and female [amygdala: +47.8 ± 21.1%; *t*-test *p* = 0.02 (**Figure 4D**); NAc: +72.2 ± 26.5%; Mann–Whitney test *p* = 0.01 (**Figure S3D**)] P rat brains. As expected, in males, the upregulation of p110δ-PI(3)K is accompanied by a reduction in TIRAP levels both in the amygdala (−13.7 ± 6.0%; *t*-test *p* = 0.02) (**Figure 4E**) and in the NAc (−16.7 ± 7.1%; *t*-test *p* = 0.02) (**Figure S3E**). In females, a significant reduction in TIRAP occurs in the amygdala (−33.2 ± 8.9%; *t*-test *p* = 0.002) (**Figure 4F**) but not in the NAc (**Figure S3F**). The results indicate that 3α,5α-THP’s activation of p110δ-PI(3)K may lead to reduced TIRAP levels in the brains of male P rats and, to some extent, in certain brain regions of female P rats.

In addition, we evaluated additional potential members of the anti-inflammatory TLR4/TRIF signaling pathway, including transcription factor cellular musculoaponeurotic fibrosarcoma (c-Maf), phosphorylated protein kinase B (pAkt), heat shock protein 70 (HSP70), and pCREB, in tissue lysates obtained from the amygdala and NAc of male and female P rats following the administration of 3α,5α-THP or vehicle control. Our findings reveal that these proteins either demonstrated no response to 3α,5α-THP or were inhibited by 3α,5α-THP, indicating that they are unlikely to play a significant role in the anti-inflammatory regulation. In the male amygdala and male NAc, c-Maf demonstrated no response to 3α,5α-THP (*p* > 0.05) (**Table 1**). In the female amygdala, c-Maf levels also showed no significant response to 3α,5α-THP (*p* > 0.05) (**Table S2**). However, in the female NAc, c-Maf levels were inhibited (−29.4 ± 6.5%, *t*-test *p* = 0.01) (**Table S2**). The levels of pAkt exhibited inhibition by 3α,5α-THP in the male amygdala (−34.2 ± 26.4%, Mann–Whitney test *p* = 0.04) and male NAc (−20.7 ± 3.5%, Mann–Whitney test *p* = 0.02) (**Table 1**). Conversely, pAkt showed no significant response to 3α,5α-THP in the female amygdala and female NAc (*p* > 0.05) (**Table S2**). 3α,5α-THP led to inhibition of HSP70 levels in the male NAc (−12.4 ± 5.1%, *t*-test, *p* = 0.04) (**Table 1**) and female amygdala (−23.0 ± 7.1%, Mann–Whitney test, *p* = 0.02) (**Table S2**). However, HSP70 levels did not significantly change in the male amygdala (**Table 1**) and female NAc (**Table S2**) in response to 3α,5α-THP. 3α,5α-THP resulted in the inhibition of pCREB levels in the male NAc (−39.0 ± 2.4%, Mann–Whitney test *p* = 0.002) (**Table 1**) and female NAc (−13.0 ± 3.5%, *t*-test *p* = 0.04) (**Table S2**). In contrast, pCREB levels showed no significant change in the male amygdala (**Table 1**) or female amygdala (**Table S2**) in response to 3α,5α-THP.

### 3.4 3α,5α-THP enhances TLR4 and TRIF translocation to endosomes and promotes endosomal Rab7 levels without affecting EEA1

To better understand the anti-inflammatory effects of 3α,5α-THP on the endosomal TLR4/TRIF signaling pathway, we isolated endosomes from the amygdala of male P rats after administering either 3α,5α-THP (*n* = 10) or a vehicle control (*n* = 10) and examined the levels of endosomal proteins, TLR4, TRIF, TLR3, Ras-related protein Rab-7 (Rab7), and EEA1 (55).

**Figure 5:**
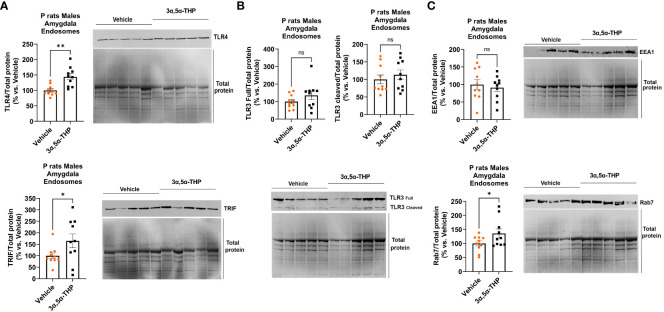
3α,5α-THP increases TLR4 and TRIF accumulation in endosomes and elevates endosomal Rab7 levels without impacting EEA1 in the male P rat amygdala. After intraperitoneal administration of either 3α,5α-THP (10 mg/kg) or a vehicle control to male P rats, we isolated endosomes from amygdala and analyzed the levels of TLR4, TRIF, TLR3, Rab7, and EEA1 within the endosomes. **(A)** 3α,5α-THP significantly increased the accumulation of TLR4 (+43.9 ± 11.3%; *t*-test: *t* = 3.89, df = 18, *n* = 10, *p* = 0.001) and TRIF (+64.8 ± 32.8%; *t*-test: *t* = 1.98, df = 18, *n* = 10, *p* = 0.03) within endosomes. **(B)** In contrast, 3α,5α-THP had no significant impact on the endosomal accumulation of either full-length TLR3 (*t*-test: *t* = 1.32, df = 18, *n* = 10, *p* = 0.10) or cleaved TLR3 (Mann–Whitney test: *U* = 40, *n* = 10, *p* = 0.48). **(C)** Additionally, 3α,5α-THP induced a significant elevation in Rab7 levels within endosomes (+35.8 ± 18.4%; *t*-test: *t* = 1.95, df = 18, *n* = 10, *p* = 0.03), while EEA1 levels showed no statistically significant change (*t*-test: *t* = 0.50, df = 18, *n* = 10, *p* = 0.31). *p < 0.05; **p < 0.01.

3α,5α-THP administration produced an increase in the accumulation of TLR4 (+43.9 ± 11.3%; *t*-test *p* = 0.001) and TRIF (+64.8 ± 32.8%; *t*-test *p* = 0.03) within endosomes (**Figure 5A**), but did not affect the total levels of TLR4 and TRIF in the whole-tissue lysates of male P rat brains (**Table 1**). In contrast, 3α,5α-THP administration had no significant effect on both full-length TLR3 or cleaved TLR3 endosomal accumulation (**Figure 5B**). These results suggest that 3α,5α-THP likely impacts the endosomal TLR4/TRIF signaling pathway, while having no significant effect on the endosomal TLR3/TRIF signaling pathway.

Furthermore, we observed that 3α,5α-THP induced a rise in Rab7 levels within endosomes (+35.8 ± 18.4%; *t*-test *p* = 0.03), but there was no significant effect on EEA1 levels (**Figure 5C**). These findings imply that 3α,5α-THP promotes the transition between early endosomes (EEA1) and late endosomes (Rab7).

### 3.5 3α,5α-THP and 3α,5α-THDOC elevate IL-10 levels in RAW264.7 cells following imiquimod-induced reduction

**Figure 6:**
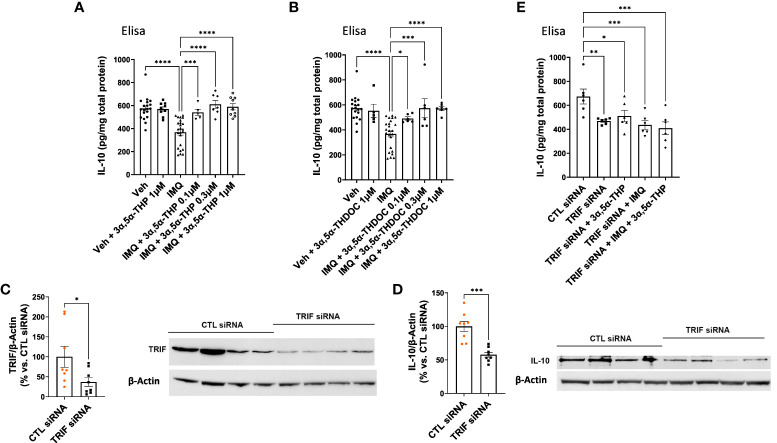
Modulation of IL-10 levels by 3α,5α-THP and 3α,5α-THDOC in RAW264.7 cells. We explored the potential impact of 3α,5α-THP (1.0 µM) and 3α,5α-THDOC (1.0 µM) on IL-10 levels within non-activated RAW264.7 cells (non-treated with TLR7 agonist imiquimod). Under these conditions, neither **(A)** 3α,5α-THP nor **(B)** 3α,5α-THDOC demonstrated statistically significant effects on IL-10 levels (Fisher’s test: *p* > 0.05). In contrast, the TLR agonist imiquimod (30 µg/mL) reduced IL-10 levels by -36.0 ± 5.9% [Fisher’s test: *p* < 0.0001, *n* = 19; see **(A)** and **(B)**]. Both **(A)** 3α,5α-THP (0.1, 0.3, and 1.0 µM) [one-way ANOVA: *F* (5, 57) = 15.06, *p* < 0.0001] and **(B)** 3α,5α-THDOC (0.1, 0.3, 1.0 µM) [one-way ANOVA: *F* (5, 58) = 9.91, *p* < 0.0001] restored IL-10 levels to those observed in the vehicle control group (Veh). To elucidate the role of the TRIF-dependent TLR4 pathway, we conducted TRIF siRNA transfection experiments in RAW264.7 cells and the levels of TRIF and IL-10 were examined using an immunoblotting assay. The reduction of **(C)** TRIF (−62.9 ± 28.2%; *t*-test: *t* = 2.23, df = 14, *n* = 8, *p* = 0.04) through TRIF siRNA transfection (20 nM, 72 h) led to a significant decrease in **(D)** IL-10 levels (−42.3 ± 8.4%; *t*-test: *t* = 5.05, df = 14, *n* = 8, *p* = 0.0002). This effect was further confirmed by ELISA, showing a decrease in **(E)** IL-10 levels [one-way ANOVA: *F* (4, 25) = 5.37, *p* = 0.003; Fisher’s test: −30.4 ± 9.3%, *n* = 6, *p* = 0.004] following TRIF downregulation. Treatment with 3α,5α-THP (1.0 µM) did not reverse the reduced IL-10 levels (Fisher’s test: *p* = 0.52, *n* = 6). Similarly, imiquimod (30 µg/mL) alone or in combination with 3α,5α-THP (1.0 µM) did not alter the decreased level of IL-10 (Fisher’s test: *p* = 0.61 and *p* = 0.36, respectively, *n* = 6). *p < 0.05; **p < 0.01; ***p < 0.001; ****p < 0.0001.

In our previous studies, we demonstrated the inhibitory effects of 3α,5α-THP, but not 3α,5α-THDOC on the activation of inflammatory TLR4 signals and the subsequent production of pro-inflammatory cytokines and chemokines, such as tumor necrosis factor alpha, high mobility group box 1, and monocyte chemoattractant protein-1 in the RAW264.7 monocyte/macrophage cell line (6). As part of our ongoing investigation, we examined the impact of 3α,5α-THP and 3α,5α-THDOC on IL-10 levels. It has been previously shown that TLR7 activation inhibits IL-10 expression in immune cells (56). Therefore, RAW264.7 cells were activated with the TLR7 agonist imiquimod (30 µg/mL) and treated with varying concentrations of 3α,5α-THP (0.1, 0.3, and 1.0 µM) or 3α,5α-THDOC (0.1, 0.3, and 1.0 µM), or a vehicle control. We also investigated the effects of 3α,5α-THP and 3α,5α-THDOC on the levels of IL-10 in the absence of TLR7 activation. Neither 3α,5α-THP (1.0 µM) nor 3α,5α-THDOC (1.0 µM) exhibited any significant effects (Fisher’s test: *p* > 0.05) on IL-10 levels in non-activated RAW264.7 cells (cells not treated with imiquimod) (**Figures 6A, B**). Following activation of RAW264.7 cells by imiquimod, both 3α,5α-THP (one-way ANOVA, *p* < 0.0001) and 3α,5α-THDOC (one-way ANOVA, *p* < 0.0001) led to elevated IL-10 levels. Imiquimod activation of RAW264.7 cells reduced IL-10 levels by −36.0 ± 5.9% (Fisher’s test: *p* < 0.0001) compared to the vehicle control. 3α,5α-THP restored IL-10 levels (Fisher’s test: 0.1 µM: +30.2 ± 9.6%, *p* = 0.0006; 0.3 µM: +42.3 ± 8.5%, *p* < 0.0001; 1.0 µM: +38.6 ± 7.5%, *p* < 0.0001) to levels statistically equivalent to control levels (**Figure 6A**). All three concentrations of 3α,5α-THDOC demonstrated a significant increase in IL-10 levels (Fisher’s test: 0.1 µM: +21.6 ± 8.6%, *p* = 0.02; 0.3 µM: +35.9 ± 10.5%, *p* = 0.0001; 1.0 µM: +35.4 ± 8.0%, *p* < 0.0001). Both 0.3 µM and 1.0 µM concentrations of 3α,5α-THDOC demonstrated comparable effects on IL-10 levels, and the increases in IL-10 levels by 3α,5α-THDOC were statistically indistinguishable from the control IL-10 levels (**Figure 6B**).

### 3.6 Role of TRIF-dependent TLR4 signaling pathway in IL-10 production: evidence from siRNA transfection experiment in RAW264.7 cells

To validate the role of the TRIF-dependent TLR4 signaling pathway in IL-10 production, we transfected RAW264.7 cells with TRIF siRNA (20 nM, 72 h), and measured IL-10 levels using an immunoblotting assay. The findings showed that the downregulation of TRIF (−62.9 ± 28.2%; *t*-test, *p* = 0.04) resulted in a decrease in IL-10 levels (−42.3 ± 8.4%, *t*-test, *p* = 0.0002) (**Figures 6C, D**). ELISA results further confirmed that the downregulation of TRIF resulted in a decrease in IL-10 levels (one-way ANOVA, *p* = 0.003; Fisher’s test: −30.4 ± 9.3%, *p* = 0.004) (**Figure 6E**). Importantly, 3α,5α-THP (1.0 µM) did not alter the reduced level of IL-10 (Fisher’s test: *p* = 0.52). Furthermore, imiquimod (30 µg/mL) alone (Fisher’s test: *p* = 0.61) or 3α,5α-THP (1.0 µM) in combination with imiquimod did not restore the decreased level of IL-10 (**Figure 6E**) in the cells treated with TRIF siRNA. These results provide strong support for the role of TRIF, a key adaptor for endosomal TLR4, in the production of IL-10 as well as its enhancement by 3α,5α-THP.

## 4 Discussion

**Figure 7:**
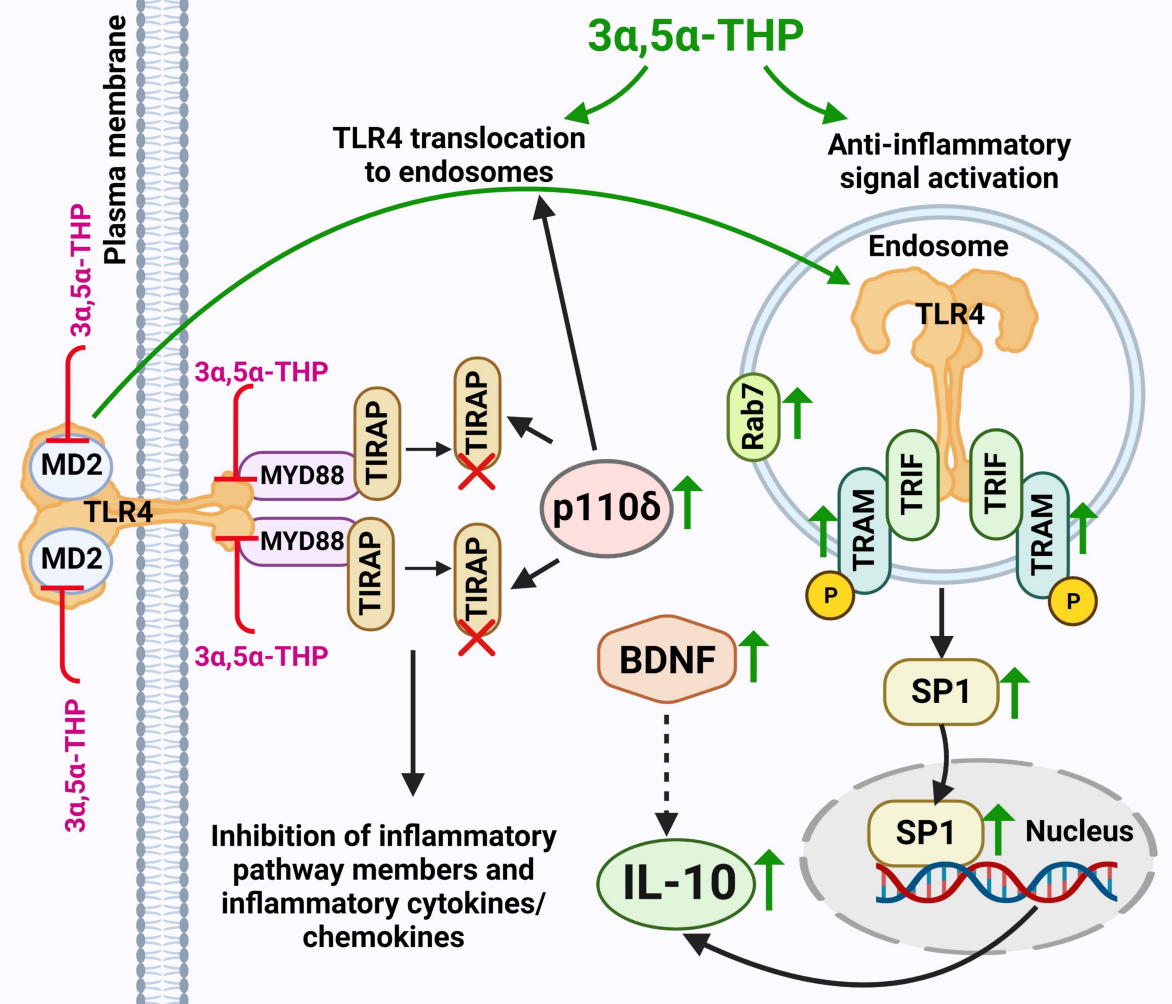
Schematic model of 3α,5α-THP induction of endosomal TLR4-TRIF anti-Inflammatory signaling and the subsequent elevation of IL-10 levels. 3α,5α-THP facilitates the transition of TLR4 from the TIRAP-MyD88-associated plasma membrane complex to an endosomal TRAM-TRIF complex, triggering the activation of the endosomal anti-inflammatory TLR4-TRIF signal and subsequent induction of IL-10 production. Mechanistically, 3α,5α-THP upregulates the p110δ isoform of PI(3)K, which, in turn, triggers the degradation of TIRAP and the release of TLR4 from the TIRAP-MyD88-associated plasma membrane complex, facilitating TLR4 translocation to endosomes. This process is supported by studies by Aksoy et al. (2012) (29) and Siegemund and Sauer (2012) (27). The translocation of TLR4 to endosomes may be facilitated by its adaptors TRAM or TRIF, as discussed in Kagan’s article (2012) (59). Additionally, the involvement of 3α,5α-THP in TIRAP degradation was documented by Murugan et al. (2019) (5). Notably, the release of TLR4 from the TIRAP-MyD88-associated plasma membrane complex could also be attributed to direct 3α,5α-THP-induced inhibition of the binding between TLR4 and MyD88, and TLR4 and MD2, as observed in Balan et al.’s studies (2019, 2021) (6, 7). Furthermore, both the inhibition of binding and TIRAP degradation led to the suppression of inflammatory TLR4 pathway components, along with inflammatory cytokines and chemokines, as indicated by studies by Balan et al. (2019, 2021, 2022, 2023) (6–8, 10) and Murugan et al. (2019) (5). 3α,5α-THP activates the anti-inflammatory endosomal TLR4-TRIF pathway by triggering an increase in phosphorylated (p) or activated TRAM, which acts as a specific marker for TLR4-TRIF pathway activation (25). The model further encompasses the 3α,5α-THP-induced enhanced presence of transcription factor SP1, leading to an increase in the production of anti-inflammatory cytokine IL-10. Additionally, 3α,5α-THP upregulates the levels of BDNF, which might further amplify IL-10 production and release (30–32). Furthermore, 3α,5α-THP stimulates the accumulation of endosomal Rab7, a process that potentially exerts a significant impact on the equilibrium between pro-inflammatory and anti-inflammatory TLR4 signaling pathways. An increase in protein levels is indicated by a green up-arrow. Protein degradation is indicated by a red X. Inhibition of protein–protein binding is indicated by a red T sign. The schematic figure was created with BioRender.com.

This work offers evidence for the potential of 3α,5α-THP in enhancing IL-10 levels through elevated endosomal TLR4-TRIF anti-inflammatory signals (**Figure 7**). However, these effects were specific to male P rat brains and were not observed in the female P rat brains. No direct statistical comparison was made between males and females, since we tested them in separate experiments. However, it is noteworthy that we found effects of 3α,5α-THP on virtually every component of the TRIF-dependent TLR4 pathway in the tissues from male, but not female rats, thereby independently replicating the sex-specific effects in multiple separate experiments within the study. In male P rats, administering 3α,5α-THP led to a significant increase in IL-10 levels within both the amygdala and NAc. Similar effects were observed following 3α,5α-THDOC administration in male P rats. This marks the first demonstration of 3α,5α-THP enhancement of IL-10 levels in the brain through the modulation of TLR4-TRIF signaling.

The implications of IL-10’s role are substantial at the central nervous system (CNS) level (34). IL-10 plays a critical role in inhibiting the production of pro-inflammatory cytokines by microglia, thus safeguarding astrocytes from excessive inflammation (60, 61). Additionally, IL-10 has been shown to enhance the production of transforming growth factor β by astrocytes (62). In neurons, IL-10 receptor signaling has been linked to increased cellular survival (58, 63) and the regulation of adult neurogenesis (64, 65). This positions IL-10 as a pivotal mediator of communication among microglia, astrocytes, and neurons. It is worth noting that besides its established role in regulating immune interactions in the CNS, numerous studies directly link impaired IL-10 production or signaling to neurological disorders in patients and animal models. These conditions encompass a range of indications, from neuropathic pain (66) to multiple sclerosis (67), Alzheimer’s disease (68), and Parkinson’s disease (57). The involvement of 3α,5α-THP in upregulating IL-10, coupled with its previously recognized inhibitory effects on TLR-mediated pro-inflammatory signaling and the production of cytokines and chemokines, underscores the broader anti-inflammatory role played by this neurosteroid (5–8, 10). In contrast, 3α,5α-THDOC mimicked the effects of 3α,5α-THP on the enhancement of IL-10 levels, but not in the inhibition of TLR4 activation in cultured macrophages from male donors (10). These differences highlight the structural specificity of each neurosteroid in the regulation of distinct TLR-mediated immune pathways.

In addition, our findings revealed a substantial elevation in BDNF levels within the amygdala and NAc of male P rats following 3α,5α-THP administration, suggesting potential indirect neurotrophic effects of 3α,5α-THP (**Figure 7**). Notably, a similar effect of 3α,5α-THP on BDNF levels in the amygdala of male rats was previously reported in the study by Naert et al. (2007) (69). Furthermore, a correlation between decreased cerebrocortical 3α,5α-THP levels and the decline in hippocampal BDNF levels in male rats was established by Nin et al. (2011) (70) and Almeida et al. (2020) (71). Almeida et al. also successfully restored BDNF levels upon administration of 3α,5α-THP. In a clinical context, patients anesthetized with alfaxalone, an analog of 3α,5α-THP, during hip replacement surgery exhibited higher serum BDNF levels compared to those anesthetized with propofol and sevoflurane (72). Moreover, several studies have suggested a potential interplay between BDNF and IL-10 production and release (30–32), suggesting a possible connection between neurotrophic and anti-inflammatory signaling pathways.

In contrast, the effects of 3α,5α-THP in females differed. 3α,5α-THP did not significantly influence IL-10 and BDNF expression in the amygdala or NAc of female P rats. The data align with Almeida et al.’s (2020) (71) observations that 3α,5α-THP modulated BDNF levels in male rat brains, but not in female rat brain. Our findings have demonstrated distinct and sex-specific outcomes of 3α,5α-THP’s influence on the TRIF-dependent TLR4 signaling pathway within the P rat brain. Specifically, in males, 3α,5α-THP activated the TLR4/TRIF signaling pathway, while in females, it inhibited the TLR4/TRIF signaling pathway. Since the activation of the TLR4/TRIF pathway and the concurrent elevation in IL-10 levels was present in males and absent in females, it implies a potential association between TLR4/TRIF signaling and IL-10 production specifically in males. Thus, in males, the administration of 3α,5α-THP triggered a significant elevation in phosphorylated (activated) TRAM levels, observed both in the amygdala and in the NAc. The activation of TRAM serves as a direct indicator of the activation of the TRIF-dependent TLR4 pathway (25, 26). In contrast, in female P rats, the administration of 3α,5α-THP resulted in the suppression of phosphorylated TRAM levels in both the amygdala and the NAc. These differences raise questions about the underlying factors contributing to these sex-specific responses. Considering the complex interplay of hormonal, genetic, and epigenetic factors influencing neuroinflammation and trophic signaling, it is reasonable to assume that 3α,5α-THP’s effects are mediated by sex-dependent mechanisms (10, 73–79). In addition, the distinct impact of 3α,5α-THP on TLR4 signaling in males and females could also arise from sex-specific protein–protein interactions upon TLR4 activation, leading to potential alterations in the TLR4 signaling pathways (80–83). Additional research is essential to unravel the molecular, hormonal factors and/or sex-specific protein–protein interactions underlying these sex-specific effects of 3α,5α-THP on the TLR4 anti-inflammatory signaling and IL-10 and BDNF expression.

To enhance our understanding of the anti-inflammatory effects of 3α,5α-THP on the endosomal TLR4/TRIF signaling pathway, we assessed the levels of TLR4, TRIF, TLR3, Rab7, and EEA1 within isolated endosomes from the amygdala of male P rats. These rats were exposed to either 3α,5α-THP or a vehicle control. Notably, we observed significant increases in both TLR4 and TRIF within the endosomes. In contrast, the administration of 3α,5α-THP seemed to have limited impact on the accumulation of TLR3 within the endosomes. These results suggest that 3α,5α-THP might trigger the transfer of TLR4 from the plasma membrane to endosomes, a process that probably depends on adaptor proteins TRIF and/or TRAM. This aligns with the established role of adaptor proteins TIRAP (at the plasma membrane) and TRAM (in endosomes) in governing TLR4 compartmentalization (59). Furthermore, we observed an elevation in p110δ-PI(3)K levels in the brain following 3α,5α-THP administration. Notably, p110δ-PI(3)K is known to facilitate the transition of TLR4 from an initial TIRAP-MyD88-associated complex at the plasma membrane (associated with proinflammatory mediators) to a subsequent endosomal TRAM-TRIF complex (associated with anti-inflammatory mediators like IL-10) (**Figure 7**). Mechanistically, p110δ-PI(3)K partly acts by reducing the presence of the TIRAP-anchoring lipid phosphatidylinositol 4,5-bisphosphate (PtdIns(4,5)P2) at the plasma membrane, which triggers TLR4 endocytosis via Ca^2+^ mobilization. This turnover of PtdIns(4,5)P2 leads to the release of TIRAP into the cytoplasm, where it undergoes degradation by calpains and the proteasome. Deactivation of p110δ-PI(3)K shifts the balance towards early proinflammatory TLR4 signaling (27, 29).

Additionally, our study examined the progression of endosomal maturation by assessing the levels of EEA1 (a marker of the early endosome stage) and Rab7 (a marker of the late endosome stage) (55) within endosomes isolated from the amygdala of male P rats that were treated with 3α,5α-THP or a control vehicle. Notably, we observed an elevation in Rab7 levels within the endosomes following 3α,5α-THP administration. In contrast, the levels of EEA1 remained unaffected by the 3α,5α-THP treatment. These results suggest a potential role for 3α,5α-THP in facilitating the transition between early endosomes, marked by EEA1, and the subsequent stage characterized by the presence of Rab7 as a marker of late endosomes, a process that likely influences the balance between proinflammatory and anti-inflammatory TLR4 signaling.

TRIF siRNA transfection in mouse RAW264.7 macrophages confirmed the pivotal role of the TRIF-dependent TLR4 signaling pathway in IL-10 production. The downregulation of TRIF led to a significant decrease in IL-10 levels and prevented 3α,5α-THP enhancement of IL-10 levels, highlighting TRIF’s critical function as a key adaptor in endosomal TLR4-mediated IL-10 production. Additionally, the study delved into the interplay between pro-inflammatory TLR7 activation and TRIF-dependent anti-inflammatory TLR4 activation, along with the effects of 3α,5α-THP and 3α,5α-THDOC on IL-10 expression using RAW264.7 cells. When these cells were activated by the TLR7 agonist imiquimod, there was a noticeable reduction in IL-10 levels, consistent with findings by Chodisetti et al. (2020) (56). However, treatment with different concentrations (0.1, 0.3, and 1.0 µM) of 3α,5α-THP and 3α,5α-THDOC effectively reversed the imiquimod-induced reduction in IL-10. Importantly, these neurosteroids did not significantly affect non-activated RAW264.7 cells. It is noteworthy that our earlier research demonstrated the ability of both neurosteroids to inhibit inflammatory TLR7 activation in female human macrophages, with 3α,5α-THP also inhibiting inflammatory TLR7 activation in RAW264.7 cells (7, 10). This dual mechanism involving both TLR7 and TLR4/TRIF signaling provides valuable insights into the therapeutic potential of these neurosteroids for modulating immune responses and controlling inflammation.

The potential involvement of SP1, c-Maf, p110δ-PI(3)K, TIRAP, Akt, HSP70, and CREB (27, 29, 84–88) in the TLR4 anti-inflammatory signaling pathways and their potential modulation by 3α,5α-THP, particularly in the male P rat brains, was also examined. Our investigation extended to the transcription factors SP1 and c-Maf, known to influence IL-10 production (54, 86). Notably, our results revealed elevated levels of SP1 in both male and female P rat brains following 3α,5α-THP treatment. These increases in SP1 levels suggests that the role of this transcription factor could potentially extend beyond its established regulation of IL-10 expression, as observed in the male P rat brain. Moreover, Xu et al. (2016) (89) have suggested that SP1 might also play a role in regulating other genes. Therefore, it is plausible that 3α,5α-THP could exert a broader influence on gene expression by upregulating SP1.

We examined the activation of p110δ-PI(3)K, a key participant in the anti-inflammatory TLR4/TRIF signaling pathway, influencing the degradation of TIRAP, a central adaptor engaged in inflammatory TLR4/MyD88 signaling (27, 29). Our results indicate that 3α,5α-THP significantly enhances p110δ-PI(3)K levels in both male and female P rat brains. Consistently, in males, the elevation of p110δ-PI(3)K is accompanied by a concurrent reduction in TIRAP levels, observed in both the amygdala and the NAc regions. A similar reduction in TIRAP is observed in the amygdala of females, albeit without a significant change in the NAc. This reduction in TIRAP levels may consequently lead to a decrease in TIRAP-dependent inflammatory TLR4/MyD88 signaling, aligning with previous observations (27, 29). Additionally, the study by Murugan et al. (2019) (5) demonstrated 3α,5α-THP’s involvement in TIRAP and TLR2 degradation in macrophages and microglial cells.

Prior research has shown that the activation of signaling pathways involving Akt in humans leads to a shift in microglia polarization from the proinflammatory M1 phenotype to the anti-inflammatory M2 phenotype (84). However, our findings indicate that 3α,5α-THP inhibits the activation of Akt, as evidenced by decreased levels of phosphorylated (activated) Akt in both the amygdala and the NAc of male P rats. This suggests that Akt activation may not play a role in the anti-inflammatory signaling pathway. Notably, an association has been previously identified between the Akt activation and increased astrocyte reactivity (90). Therefore, potential therapeutic approaches utilizing 3α,5α-THP to inhibit Akt activation could offer avenues for addressing the negative impacts of astrogliosis (91).

Previous studies have highlighted that the anti-inflammatory compounds resveratrol (87), nobiletin (92), and quercitrin (88) exert their neuroprotective effects through signaling pathways involving CREB and BDNF. Nevertheless, we found that phosphorylated (activated) CREB levels decrease in the NAc, while BDNF levels increase in both the NAc and the amygdala of male P rats after 3α,5α-THP administration. These results imply that the CREB and BDNF pathways operate independently. Unlike the BDNF pathway, it appears that the CREB pathway does not contribute to the anti-inflammatory responses. However, it is important to note that 3α,5α-THP might exert a transient impact on CREB concentrations. Thus, intravenous 3α,5α-THP administration triggers a rapid but temporary rise in CREB at 5 min, followed by a significant decline relative to baseline by 15 min in plasma (93). Moreover, it is worth mentioning that CREB’s role extends beyond BDNF production, encompassing the generation of inflammatory mediators (94). Our prior studies have demonstrated that 3α,5α-THP substantially inhibits phosphorylated/activated CREB levels in RAW264.7 cells and human macrophages activated by TLR4 agonist lipopolysaccharide (6, 7).

In conclusion, these findings provide strong evidence that 3α,5α-THP and 3α,5α-THDOC enhance IL-10 levels through endosomal TLR4-TRIF anti-inflammatory signals, in male rat brains. This amplification of IL-10, a critical mediator of immune homeostasis, holds significant implications for the CNS and the periphery, glial, and neuronal cells as well as immune cells. Furthermore, the observed elevation in BDNF levels within male rat brains suggests that 3α,5α-THP may exert neurotrophic effects. The ability of these endogenous neurosteroids to enhance IL-10 production in male P rat brains and mouse macrophages positions them as valuable candidates for therapeutic intervention aimed at immune and neuroimmune disorders. These results together with known inhibitory effects of 3α,5α-THP on TLR pro-inflammatory pathways in both male and female P rat brain as well as mouse and human macrophages (5–10) contribute to the broader understanding of 3α,5α-THP’s anti-inflammatory and neuroprotective roles. Further research is necessary to unravel the underlying molecular mechanisms and to elucidate the sex-specific differences in 3α,5α-THP’s effects on the IL-10 pathway, ultimately paving the way for potential clinical applications in promoting immune homeostasis and mitigating inflammation- and neuroinflammation-related conditions.

## Data availability statement

The raw data supporting the conclusions of this article will be made available by the authors, without undue reservation.

## Ethics statement

The animal study was approved by Institutional Animal Care and Use Committee at the University of North Carolina, School of Medicine. The study was conducted in accordance with the local legislation and institutional requirements.

## Author contributions

IB: Conceptualization, Data curation, Formal analysis, Investigation, Validation, Writing – original draft, Writing – review & editing. AG: Data curation, Investigation, Writing – review & editing. TKO: Data curation, Investigation, Writing – review & editing. ALM: Conceptualization, Funding acquisition, Project administration, Resources, Supervision, Validation, Writing – original draft, Writing – review & editing.
