# Supplementary material for: Neurosteroid [3α,5α]-3-hydroxy-pregnan-20-one enhances IL-10 production via endosomal TRIF-dependent TLR4 signaling pathway
Source: Front Endocrinol (Lausanne). 2023 Dec 21;14:1299420. doi: 10.3389/fendo.2023.1299420 (PMC10765172; doi:10.3389/fendo.2023.1299420)
Supplement: Supplementary file 1 [file DataSheet_1.docx]

**Supplementary Materials.**

**Table S1. Clonality, host species, and dilutions of primary antibodies used in immunoblotting**.

| **Target** | **Catalog #** | **Commercial supplier** | **Clonality** | **Host** | **Dilution** |
| --- | --- | --- | --- | --- | --- |
| IL-10 | LS-C756365 | LifeSpan BioSciences, Lynnwood, WA, USA | Polyclonal | Rabbit | 1:300 |
| BDNF | OSB00017W | Thermo Fisher Scientific, Waltham, MA, USA | Polyclonal | Rabbit | 1:1000 |
| pTRAM (phospho-TICAM2) | PTRAM-140AP | FabGennix International, Frisco, TX, USA | Polyclonal | Rabbit | 1:750 |
| SP1 | sc-17824 | Santa Cruz Biotechnology, Santa Cruz, CA, USA | Monoclonal | Mouse | 1:100 |
| PI3 Kinase p110δ | 34050 | Cell Signaling Technology, Danvers, MA, USA | Monoclonal | Rabbit | 1:1000 |
| TIRAP | LS-C747701 | LifeSpan BioSciences, Lynnwood, WA, USA | Polyclonal | Rabbit | 1:500 |
| TRIF (TICAM-1) | 657102 | BioLegend, San Diego, CA, USA | Monoclonal | Mouse | 1:500 |
| TLR4 | sc-293072 | Santa Cruz Biotechnology, Santa Cruz, CA, USA | Monoclonal | Mouse | 1:500 |
| TLR3 | PA5-88251 | Thermo Fisher Scientific, Waltham, MA, USA | Polyclonal | Rabbit | 1:500 |
| c-Maf | sc-293420 | Santa Cruz Biotechnology, Santa Cruz, CA, USA | Monoclonal | Mouse | 1:200 |
| Phospho-Akt (Ser473) | 4060 | Cell Signaling Technology, Danvers, MA, USA | Monoclonal | Rabbit | 1:2000 |
| HSP70 | sc-24 | Santa Cruz Biotechnology, Santa Cruz, CA, USA | Monoclonal | Mouse | 1:200 |
| Phospho-CREB (Ser133) | 9198 | Cell Signaling Technology, Danvers, MA, USA | Monoclonal | Rabbit | 1:1000 |
| EEA1 | 2411 | Cell Signaling Technology, Danvers, MA, USA | Polyclonal | Rabbit | 1:1000 |
| Rab7 | 2094 | Cell Signaling Technology, Danvers, MA, USA | Polyclonal | Rabbit | 1:1000 |
| β-Actin | 66009-1-Ig | Proteintech Group, Rosemont, IL, USA | Monoclonal | Mouse | 1:3000 |

**Table S2. Evaluated signaling pathway members that demonstrated no response to 3α,5α-THP or they are inhibited by 3α,5α-THP in the whole tissue lysates of the amygdala and nucleus accumbens (NAc) of female P rats.**

| **Pathway members** | **3α,5α-THP vs. Vehicle in Female P Rats**  **t-test (t, df, p)/ Mann Whitney test (U, p, n)** | |
| --- | --- | --- |
|  | **Amygdala** | **NAc** |
| TLR4 | No difference  t=0.18, df=18, p=0.43 | No difference  t=0.64, df=18, p=0.26 |
| TRIF | Inhibition: -14.0±6.9%  t=1.75, df=18, p=0.049***** | Inhibition: -17.0±6.2%  t=1.89, df=18, p=0.04***** |
| c-Maf | No difference  t=0.76, df=18, p=0.23 | Inhibition: -29.4±6.5%  t=2.55, df=18, p=0.01***** |
| pAkt | No difference  t=0.13, df=18, p=0.45 | No difference  t=0.77, df=18, p=0.23 |
| HSP70 | Inhibition: -23.0±7.1%  U=18.5, p=0.02*****, n=10 | No difference  t=1.40, df=18, p=0.09 |
| pCREB | No difference  U=39, p=0.22, n=10 | Inhibition: -13.0±3.5%  t=1.814, df=18, p=0.04***** |

*****p<0.05


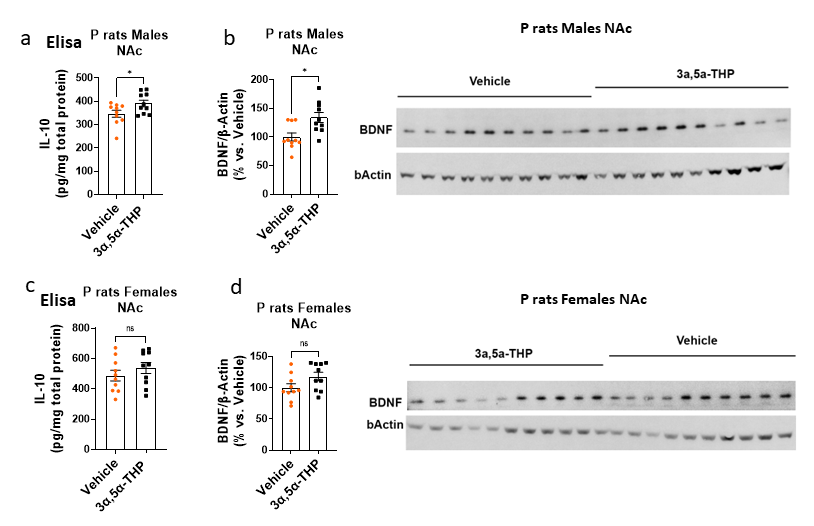


**Figure S1. 3α,5α-THP elevates IL-10 and BDNF levels in the nucleus accumbens (NAc) of male P rats.** 3α,5α-THP (10 mg/kg, IP) or a vehicle control was administered to male and female P rats, and the levels of IL-10 and BDNF were determined within the NAc. (a) In male P rats, administration of 3α,5α-THP led to a significant increase in IL-10 levels in the NAc (+12.8±5.9%; t-test: t=2.18, df=18, p=0.04). (b) Similarly, a significant elevation in BDNF levels was observed in the NAc of male P rats following 3α,5α-THP treatment (+34.2±11.2%; Mann-Whitney test: U=18.0, p=0.01, n=10). (c) In contrast, female P rats did not exhibit a significant change in IL-10 expression in the NAc following 3α,5α-THP treatment (t-test: t=0.98, df=18, p=0.34). (d) Additionally, there were no significant alterations in the levels of BDNF in the NAc of female P rats after 3α,5α-THP administration (t-test: t=1.94, df=18, p=0.07). *p<0.05.


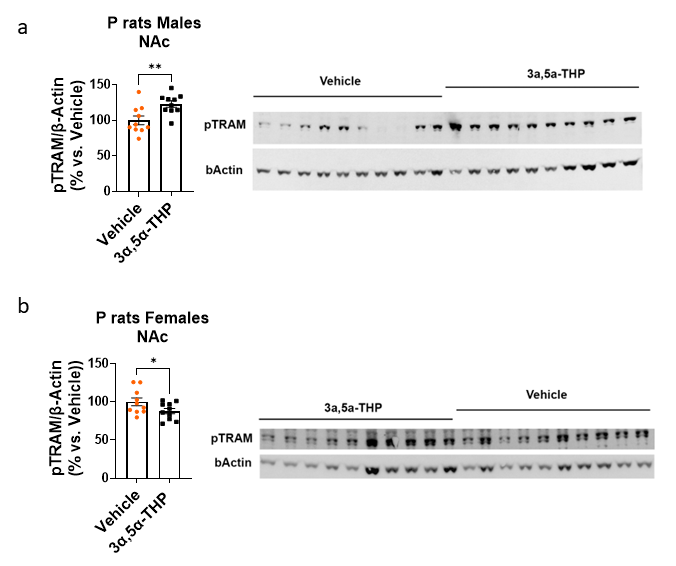


**Figure S2: Sex-specific effects of 3α,5α-THP on TRAM-dependent TLR4-TRIF signaling in the NAc of P rats: activation in males, inhibition in females.** After 3α,5α-THP (10 mg/kg, IP) or a vehicle administration to male and female P rats, we analyzed the levels of pTRAM within the NAc. (a) In males, 3α,5α-THP administration led to a significant increase in pTRAM levels (+22.9±7.5%; t-test: t=3.04, df=18, p=0.007), whereas (b) females exhibited inhibited pTRAM levels (-12.1±6.3%; t-test: t=1.94, df=18, p=0.03). *p<0.05; **p<0.01.


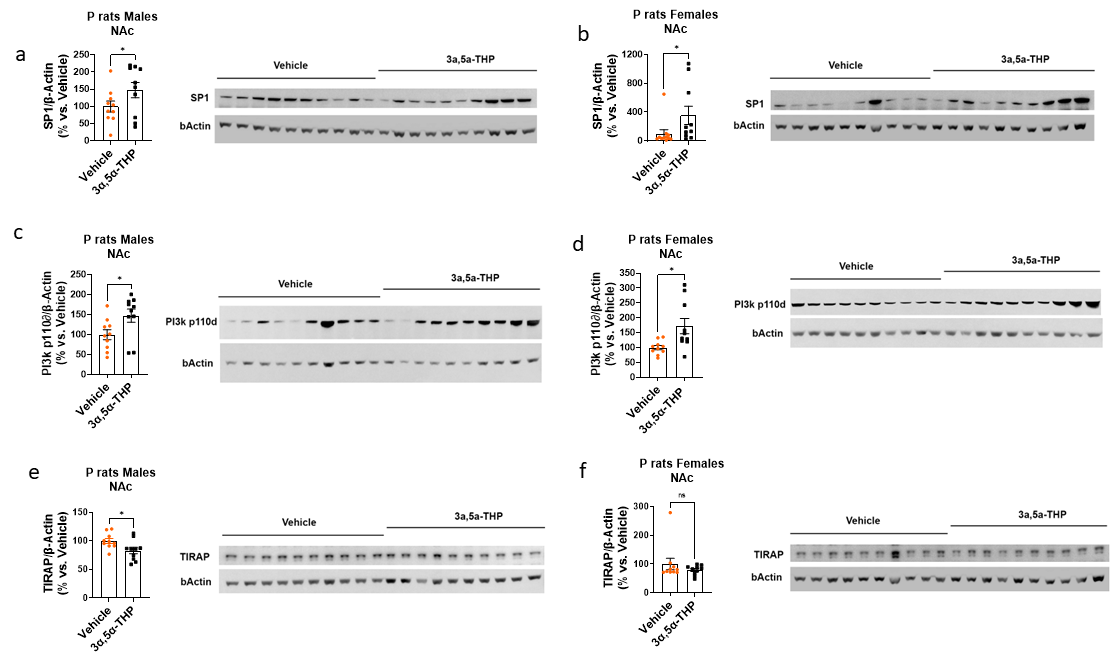


**Figure S3: 3α,5α-THP upregulates SP1 and p110δ-PI(3)K levels in the NAc of male and female P rats.** 3α,5α-THP (10 mg/kg, IP) or a vehicle control was administered to male and female P rats to assess its impact on SP1, p110δ-PI(3)K, and TIRAP levels within the NAc. 3α,5α-THP elevates SP1 levels in the NAc of both (a) male (+47.6±27.5%; t-test: t=1.73, df=18, p=0.049) and (b) female (+254.3±134.9%; Mann-Whitney test: U=22.0, p=0.02, n=10) P rats. 3α,5α-THP upregulates p110δ-PI(3)K levels in the NAc of both (c) male (+47.3±20.6%; Mann-Whitney test: U=21.5, n=10, p=0.03) and (d) female (+72.2±26.5%; Mann-Whitney test: U=17.0, p=0.01, n=10) P rats. 3α,5α-THP reduces TIRAP levels in the NAc of (e) male (-16.7±7.1%; t-test: t=2.35, df=18, p=0.02) but not (f) female (t-test: t=0.99, df=18, p=0.17) P rats. *p<0.05.
